# Ecological conditions drive pace-of-life syndromes by shaping relationships between life history, physiology and behaviour in two populations of Eastern mosquitofish

**DOI:** 10.1038/s41598-018-33047-0

**Published:** 2018-10-02

**Authors:** Giovanni Polverino, Francesca Santostefano, Carlos Díaz-Gil, Thomas Mehner

**Affiliations:** 1 Department of Biology and Ecology of Fishes, Leibniz-Institute of Freshwater Ecology and Inland Fisheries (IGB), Berlin, 12587 Germany; 2 Centre for Evolutionary Biology, School of Biological Sciences, The University of Western Australia, Perth, 6009 Australia; 3 Département des Sciences Biologiques, Université du Québec à Montréal, Montréal, QC H3C 3P8 Canada; 4 Department of Ecology and Marine Resources, Instituto Mediterráneo de Estudios Avanzados IMEDEA (CSIC-UIB), Esporles, 07190 Spain; 5 Laboratori d’Investigacions Marines i Aqüicultura (LIMIA), Balearic Government, Port d’Andratx, 07157 Spain

## Abstract

The pace-of-life syndrome (POLS) hypothesis predicts variation in behaviour and physiology among individuals to be associated with variation in life history. Thus, individuals on the “fast” end of POLS continuum grow faster, exhibit higher metabolism, are more risk prone, but die earlier than ones on the “slow” end. Empirical support is nevertheless mixed and modelling studies suggested POLS to vary along selection gradients. Therefore, including ecological variation when testing POLS is vastly needed to determine whether POLS is a fixed construct or the result of specific selection processes. Here, we tested POLS predictions between and within two fish populations originating from different ecological conditions. We observed opposing life histories between populations, characterized by differential investments into growth, fecundity, and functional morphology under identical laboratory conditions. A slower life history was, on average, associated with boldness (latency to emergence from a refuge), high activity (short freezing time and long distance travelled), and increased standard metabolism. Correlation structures among POLS traits were not consistent between populations, with the expression of POLS observed in the slow-growing but not in the fast-growing population. Our results suggest that POLS traits can evolve independently from one another and that their coevolution depends upon specific ecological processes.

## Introduction

Many animal populations display a pace-of-life syndrome (POLS^1^), suggesting that among-individual variation in behaviour (i.e., personality^2^) and physiology is maintained through its association with life-history traits^3,4^. Thus, POLS expands predictions from the classic life-history theory^5^ by including behaviour and physiology into an evolutionary framework, in which individuals differ from each other in the amount of resources allocated to either survival or reproduction^6^. For instance, higher metabolic rates allow bold individuals to sustain greater muscular activity and cellular machinery^7,8^, to be more successful in competing for resources and thus grow faster^4^, and reproduce earlier (“fast” POLS^9^) than their conspecifics on the “slow” end of POLS continuum^8^. Costs of this high performance are a less efficient immune system^10^ and a shorter life span^11^.

Nevertheless, recent meta-analyses suggested that the empirical support for POLS is ambiguous^12–14^. For example, high activity and exploration rates often relate to high survival of animals, but less often to high reproductive success^15^. Similarly, consistent differences in physiological traits (e.g., high and low resting metabolic rates) among animals are not always necessarily associated with higher or lower growth rates (reviewed by^16^) or with behavioural performance (reviewed by^17^). Hence, despite rapidly accumulating evidence for the existence of POLS across species, a substantial discrepancy between POLS predictions and observations remains.

Research on animal personality^2^ and behavioural syndromes^18,19^ suggests that the association between behavioural traits can be altered by selection more rapidly than previously thought^20,21^. In a given species, animal populations should especially exhibit behavioural correlations within ecological and evolutionary contexts in which those combinations are adaptive^20,22^. For example, different predator regimes across populations of three-spined sticklebacks (*Gasterosteus aculeatus*, Linnaeus 1758) affect life-history strategies and, accordingly, either strengthen advantageous combinations of behavioural traits or break apart maladaptive ones^20,21^. Nevertheless, genetic correlations between behavioural traits can act as evolutionary constraints by imposing selection on non-target traits and slow down adaptive responses^23^. Thus, knowledge of selection regimes and specific ecological contexts under which animal populations have evolved is useful to make predictions on whether personality may exist^24,25^ and whether correlations between personality traits should be expected^20,21^. In this direction, a restructuring of POLS predictions has recently suggested that correlations between POLS traits might exist only under certain environmental conditions^26,27^, as also pointed out by a modelling study^28^. The context-dependency of POLS would then explain its mixed support in the literature, especially at the within-species level^14,29^. Nevertheless, empirical tests on variation in POLS traits across environmental and ecological conditions are surprisingly rare^26^ (but see^30^).

The goal of this study was to fill this gap in the literature. Thus, we tested predictions from POLS between (i.e., comparing population means) and within (i.e., among-individuals differences) two different fish populations whose native environments differed largely from each other in abiotic and biotic factors. To do that, we used offspring from wild-caught Eastern mosquitofish (*Gambusia holbrooki*, Girard 1859) maintained under nearly identical laboratory conditions since birth. We first explored whether fish from the two populations differed on average in their life history (age and size at sexual maturity, fecundity, and functional morphology), behaviour (related to boldness and activity), and physiology (standard metabolic rate). Once we confirmed different investments in survival and reproduction between populations, we tested whether repeatable differences in traits among individuals were correlated in both populations as typically predicted by POLS. We expected that a) two fish populations that evolved under different ecological conditions would exhibit, on average, divergent life-history strategies (slow *vs* fast) that are detectable in their laboratory-reared progeny^6^; b) on average, fish from the fast-growing population would be bolder, more active, and have higher metabolic rates than slow-growing individuals^1^; and c) repeatable among-individual differences in traits are correlated within both populations.

## Methods

The experimental procedure was approved through an animal care permit (G 0074/15) granted by the Landesamt für Gesundheit und Soziales Berlin, Germany (LAGeSo). All measurements were carried out in accordance with its relevant guidelines and regulations.

### Study organism and maintenance

Fish utilized in this study were first-generation (F1) progeny of wild-caught mosquitofish (*Gambusia holbrooki*, Girard 1859) from two different freshwater bodies in Italy separated by over 600 km: Torre Castiglione^31^ and Maccarese^32^. Mosquitofish from these populations are presumably descendants of common ancestors introduced to Italy in the early 1900^33^, but inhabit very different selective environments. Environmental conditions in the Torre Castiglione’s sinkhole are extremely stable, whereby a consistent groundwater inflow ensures that water temperature fluctuates only by 3 °C over the course of the year. Flooded caves and permanent macrophytes offer refuge and feeding habitats for young fish and piscivorous aquatic species are absent (SP2 site described in^31^). Conversely, fish from the artificial pond in Maccarese are exposed to dramatic seasonal temperature fluctuations (>20 °C) and nursery areas can be found only for short periods during the year. Furthermore, mosquitofish from Maccarese have suffered from an intense size-related harvesting over the past twenty years, whereby their native pond has been partially drained multiple times every year and approximately 80% of large adult individuals have been exploited with dip nets (personal communication by the lake owner).

We sampled approximately 100 wild individuals from each population and housed them separately in 50-L aquaria, with a maximum density of 0.4 fish L^−1^, for a minimum of five months after capture as described in^34^. Then, pregnant females from each population were removed from their housing tanks and individually transferred into 10-L aquaria until giving birth, following the procedure described in^34^. The 10-L aquaria were inspected twice a day until newly-born fish were found. Soon after, the adult female was transferred back into its housing tank, while newly-born fish (approximately 20 individuals per clutch) were maintained in their native aquarium for 15 days after birth and were fed twice a day with live and frozen *Artemia salina* nauplii.

**Figure 1:**
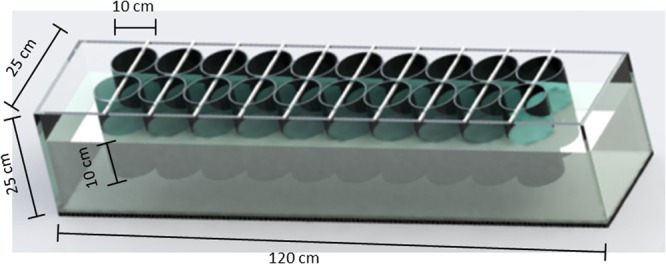
Scheme of an experimental housing tank. Each tank hosted two parallel arrays of ten transparent cylinders. Juvenile mosquitofish were housed individually in the cylinders (i.e., 20 fish per tank).

Experimental fish were then randomly selected among siblings born on the same day, with individuals showing physical anomalies and/or malformations excluded *a priori*. Experimental fish were randomly assigned to four identical experimental housing tanks (25 cm wide × 25 cm high × 120 cm long each; Fig. 1), as described in detail by^35^. Juvenile fish from the two populations were housed separately, with two experimental housing tanks (i.e., two replicas) dedicated to each population. Each experimental housing tank hosted two parallel rows of 10 transparent Plexiglas cylinders (18 cm high and 10 cm diameter) confined on their bottom surface with stainless-steel net and submerged in water for 10 cm (Fig. 1). A single fish was housed in each transparent cylinder (i.e., 20 fish per experimental housing tank) and maintained in it for the duration of the study (approximately five months).

This setup allowed visual and chemical interaction among individuals, thus eluding both behavioural biases^36^ and reduced health and longevity^37^ that are typically associated to isolation in social fish. Housing fish individually allowed identifying individuals and standardizing their housing conditions over the lifetime. We periodically randomized the position of cylinders within each experimental housing tank so that all fish experienced different neighbour compositions.

Water temperature was maintained consistently at 23 ± 0.5 °C and artificial illumination on a 12-h cycle^38^. Fish were fed at 9 a.m. and at 7 p.m with *Artemia salina* nauplii and flake food, respectively, before their maturation, while *Artemia salina* nauplii, blood worms, and flake food were used for adults.

### Experimental procedure

To estimate among-individual correlations in behaviour, physiology, and life history, we measured individual behaviour, standard metabolic rate (SMR), and life-history traits (LHTs) in a total of 80 experimental fish (40 per each population) multiple times over ontogeny. Specifically, behavioural traits related to boldness and activity were measured four times over fish ontogeny, that is, twice at onset of sexual maturation (i.e., immature fish) and twice after full sexual maturity was reached (i.e., adult stage), with trials within each stage one week apart from each other. The SMR and LHTs related to size-at-age and nutritional state were measured once per each ontogenetic stage (i.e., two measures per fish over ontogeny). LHTs related to age at sexual maturation and adult morphology and fecundity were measured at the adult stage only. Sexual maturation was detected according to the morphogenesis of the anal fin in mosquitofish males^38^. Eight experimental fish from Torre Castiglione and one fish from Maccarese died during metabolic tests due to an energy blackout overnight. This random mortality event did not bias our results and, thus, a total of 32 and 39 fish were tested at both ontogenetic stages, respectively.

### Behavioural assays

Behavioural tests were performed in a rectangular open field (60 cm long, 42 cm wide, and 30 cm high), with a capacity of 75 L (Fig. S1 in Supplementary Information, SI). As described in detail by^34^, an experimental refuge and its lid were used to acclimate each fish before initiating the test, while the squared area around the closed refuge represented the only shelter available to the fish once in the open field (Fig. S1 in SI). The open field was filled with 7 cm of conditioned water to offer natural shallow-water conditions to mosquitofish^38^, while minimizing their activity along the water column.

All behavioural tests were performed in a temperature-controlled chamber (Feutron Temperaturzelle, Feutron Klimasimulation GmbH, Greiz, Germany) between 11 a.m. and 6 p.m. from Monday to Friday. Hence, external disturbances were minimized and water temperature was maintained constantly at 23 ± 0.1 °C. A high resolution camera (Bosch Dinion HD 1080p, Bosch, Grasbrunn, Germany) was placed 1 m above the open field for a complete top view of the apparatus and it recorded the fish motion on the x-y plane. Two lateral lights provided homogeneous illumination of the open field. Fish were tested in a randomized order to exclude consistent differences in their behavioural outcome caused by hunger^39^.

Following the procedure in^35^, for each behavioural trial a single fish was netted from its transparent cylinder and assayed as follows: (1) it was gently introduced into the closed refuge and left to acclimatize for 5 min; (2) the lid of the refuge was opened and the time interval before the fish exited the refuge was assessed manually with a stopwatch (emergence latency, in s); (3) soon after the fish left the refuge, the lid was automatically closed and fish behaviour in the open field was video recorded for 10 min; and (4) the fish was transferred back into its cylinder within its original experimental housing tank. The video-tracking software (EthoVision XT Version 9.0; Noldus Information Technologies Inc.) was then utilized to calculate inactivity time (freezing time, in s), distance moved (in cm), and time spent hiding within the squared platform around the closed refuge (hiding time, in s; Fig. S1 in SI) for each fish in each video.

We interpreted short emergence latency or hiding time as fish’s willingness to take risks in exploring open spaces that were unfamiliar and potentially dangerous (i.e., boldness^2,39^), while short freezing time and long distance moved characterized high activity of individuals^2,35^.

### Standard metabolic rate

Soon after concluding the behavioural assay (at both immature and adult stages, respectively), a fish was fasted for 24 h before its SMR was measured overnight for a 12 h period (from 8 pm to 8 am). SMR was measured following standard protocols and details are presented in SI.

The mass-specific SMR (mg O_2_ kg^−1^ h^−1^) was estimated for each fish at each ontogenetic stage from the decrease in oxygen concentration over time (i.e., respiration rate^40^). Specifically, a mixture distribution composed of two normal distributions was fitted to respiration rates obtained from closed phases for a given individual (R package ‘mixtools’ v. 1.0.4^41^) and the lower mean of the two normal distributions was used as its mass-specific SMR, as per^40^. Therefore, metabolic rates measured here separated the extra oxygen consumption beyond SMRs caused by spontaneous motion activity (i.e., the higher mean of the two normal distributions) and individual costs of self-maintenance measured at a particular temperature and post-absorptive and inactive state (i.e., the true SMR^16^). To accurately measure oxygen consumption, we discarded closed phases in which oxygen did not decrease linearly over time, that is, when the R^2^ assessing the decline in oxygen over time for a given chamber was lower than 0.95. In addition, the first and last two minutes were excluded from each measure (i.e., closed phase) such that only the linear component of O_2_ degradation was captured^42^. Pilot trials determined that SMR of mosquitofish stabilized after 1 h. Thus, measurements from the first hour represented here the acclimation period to the apparatus and were not included into the estimate of SMRs.

### Life-history traits

After conclusion of the metabolic assay, fish were anesthetized in a solution of 2-phenoxiethanol (0.3 mL per 250 mL H_2_O) and measured for standard size (to the nearest 0.1 mm) and body weight (to the nearest 0.01 g) at both immature and adult stages. The Fulton’s condition factor *K*^43^ (g mm^−3^ 10^4^) was then calculated as a proxy of the nutritional state of each fish at each ontogenetic stage.

Morphometry and fecundity measurements were performed on each fish at the adult stage only (Figs. S2 and S3 in SI). Particularly, the region of the fish tail devoted to locomotor performances was measured at adulthood (i.e., swimming muscle, mm^2^), according to landmarks described by^44^. Gonopodium length (mm) was measured on all adult males as per^44^, while number of eggs, their dry weight (mg), and mean dry weight per egg (mg) were measured on adult females after they were sacrificed in a concentrated solution of 2-phenoxiethanol. Details on morphometric and fecundity measurements can be found in SI.

### Statistical Analysis

Prior to all analyses, emergence latency was log-transformed, while hiding time, freezing time, and mass-specific SMR were square-root transformed to normalize error distribution. Explanatory variables age and sex were transformed as numeric variables, both coded as −0.5 (immature and females, respectively) and 0.5 (adults and males, respectively^45,46^). All models described below were fitted using restricted maximum likelihood; dependent variables were mean-centred and their variance standardized to facilitate comparison of variance components across traits^47^. Throughout, we assumed a Gaussian error distribution, which was confirmed for all response variables after visual inspection of model residuals.

As a first step we tested whether mosquitofish descending from the two populations differed in mean LHTs despite maintained under highly standardized laboratory conditions since birth. Average differences between populations were calculated for each LHT separately with linear models (LMs) in which population was included as a fixed factor and each trait as the dependent variable. Dependent variables (i.e., LHTs) used in the LMs are listed in Table 1.

**Table 1:** Mean differences between SG and FG populations for a given LHT (±SE) and estimated marginal mean differences in behavioural traits and SMR (±SE) based on univariate models.

LHTs	SG	FG	*P*
	Mean (SE)	Mean (SE)	
Age at sexual maturation (days)	60 ± 1	30 ± 1	—
Standard size at sexual maturation (mm)	22.06 ± 0.38	20.37 ± 0.10	**<0.01**
Body weight at sexual maturation (g)	0.19 ± 0.01	0.16 ± <0.01	**<0.01**
Standard size at adulthood (mm)	27.32 ± 0.55	26.62 ± 0.27	0.09
Body weight at adulthood (g)	0.39 ± 0.02	0.34 ± 0.01	**<0.01**
Swimming muscle at adulthood (mm^2^)	112.12 ± 10.35	94.27 ± 8.55	<0.01* ^**a**^
Gonopodium length at adulthood (mm; ♂)	7.66 ± 0.09	7.47 ± 0.11	0.07
Num. of eggs at adulthood (♀)	49.42 ± 5.41	60.54 ± 5.95	0.01* ^**b**^
Dry weight of eggs at adulthood (mg; ♀)	80.53 ± 8.21	99.02 ± 8.90	0.02* ^**b**^
Mean dry weight per egg at adulthood (mg; ♀)	1.75 ± 0.15	1.66 ± 0.09	0.18*^b^
**Behavioural traits**	**Mean (SE)**	**Mean (SE)**	***P***
Emergence latency (s)	−0.22 ± 0.11	0.13 ± 0.08	0.01* ^**a**^
Hiding time (s)	2.62 ± 0.94	−1.52 ± 0.75	<0.01* ^**a**^
Distance moved (cm)	100.81 ± 59.02	−35.16 ± 47.05	0.09*^a^
Freezing time (s)	−1.85 ± 0.53	1.30 ± 0.42	<0.01* ^**a**^
**SMR**	**Mean (SE)**	**Mean (SE)**	***P***
Standard metabolic rate (mg O_2_ kg^−1^ h^−1^)	2.91 ± 2.48	−3.88 ± 1.99	0.04* ^**a**^

*Refers to mean difference between populations for a given trait after accounting for differences in standard size (*a) or body weight (*b), that is, standard size or body weight were included as fixed effects into the models to account for mean differences between populations. Symbols ♂ and ♀ refer to variables measured on males and females only, respectively. Estimated marginal mean differences represent adjusted mean differences for a given behavioural trait and SMR between fish populations once the contribution of LHTs measured repeatedly over ontogeny (i.e., standard size and Fulton’s K at a given age) and other fixed effects (i.e., age, sex, and trial) is accounted for.

We then tested whether populations differed on average in behavioural traits and SMR after accounting for mean differences in LHTs, since populations differed on average in body size but SMR largely depends on an individual’s size^48^. To do that, behavioural traits and SMR were tested separately as dependent variables (i.e., emergence latency, hiding time, distance moved, freezing time, and mass-specific SMR) with a linear mixed model (LMM) with population, age, sex, standard size, Fulton’s *K*, and trial (representing a sequence of measurements performed on each individual within a given ontogenetic stage) included as fixed factors. All other LHTs measured in this study were either significantly correlated with standard size and Fulton’s *K* (Table S1 in SI) or measured for one of the two ontogenetic stages or sexes only (Table 1) and, therefore, were excluded from these general models. The individual was specified as a random effect (i.e., random intercepts) to account for repeated measures. Nevertheless, analyses were repeated without accounting for mean differences in LHTs between populations to allow for comparisons between the two approaches (data included in SI).

Within populations, we then estimated the repeatability of all traits (i.e., behaviours, mass-specific SMR, and standard size) over ontogeny of fish. Since among-individual correlations are only expected between POLS traits that are repeatable over time^1,13^, we included in our POLS models below only those traits that were found to be repeatable over ontogeny for both populations. To measure repeatability, we ran LMMs separately for each population with each of the four behavioural traits included one-by-one as the dependent variable, the individual as a random effect (i.e., random intercepts), and age, sex, and trial as fixed factors. We then used the same model structure for the dependent variables mass-specific SMR and standard size, excluding the fixed factor trial from models since those variables were not measured repeatedly within a given ontogenetic stage. Notably, mother ID (i.e., family) and tank were not modelled since univariate models indicated that there was no variation among families and tanks for most traits (Table S2 in SI). The adjusted repeatability^49^ was estimated for each trait by calculating the proportion of the total phenotypic variance not attributable to fixed effects that was explained by among-individual variance. We tested the statistical significance of fixed effects using numerator and denominator degrees of freedom (*df*) estimated from the algebraic algorithm in ASReml 3.0^50^. We used likelihood ratio tests (LRTs) to evaluate the statistical significance of random effects (i.e., to test whether a given trait was significantly repeatable over ontogeny for a given population). This χ^2^-distributed test statistic was calculated as twice the difference in log-likelihood between a model in which a target random effect was fitted versus not fitted^51^. Variances were bound to be positive, therefore probability (*P*) of a LRT applied to a variance was calculated assuming an equal mixture of *P* (χ^2^, *df* = 0) and *P* (χ^2^, *df* = 1)^52^.

As a test of POLS within populations, we estimated patterns of covariance among individuals for each population separately for traits that were found to be repeatable over ontogeny. By expanding the same structure of univariate models described above for the repeatability to multivariate LMMs, we fitted all repeatable traits together as dependent variables. Residual variances for (and covariances between) all traits were modelled. Covariances were not bound to be positive and their probability was therefore calculated assuming *P* (χ^2^, *df* = 1). LRTs involving one variance and one covariance were tested assuming an equal mixture of *P* (χ^2^, *df* = 1) and *P* (χ^2^, *df* = 2).

As a final step, we tested whether the structure of among-individual correlations differed between populations, that is, whether the two populations differed in POLS. To do that, we constrained each pairwise covariance to be the same between the two populations. We then applied a LRT to compare the unconstrained model to the one where corresponding covariances between populations were constrained to be the same. Additionally, for each population the full model (where covariances were estimated) was compared to one where all covariance elements were constrained to zero as an overall test of among-trait covariance (i.e., POLS structure).

Data exploration and mean comparisons between populations were performed in R-3.1.1 version^53^, while repeatabilities and covariances among traits were estimated in ASReml 3.0^50^. All data associated with this manuscript are available in the Figshare repository at https://figshare.com/s/814abf5272f6902ef304.

## Results

Overall, we found that mosquitofish descending from separate wild populations differed in mean LHTs, despite being maintained under highly standardized laboratory conditions since birth (Table 1). Hence, sexual maturation started synchronically in all fish after either one month (fast-growing population from Maccarese’s pond; FG) or two months (slow-growing population from Torre Castiglione’s sinkhole; SG) since birth. Accordingly, FG fish grew faster than SG fish and FG females were also more fecund (i.e., higher number of eggs and their dry weight) than their SG counterparts (Table 1). On the contrary, SG fish reached larger sizes at adulthood and developed larger propulsion-devoted muscles than FG ones (Table 1). As a consequence, the whole body shape differed on average between the two fish populations (Fig. S4 in SI).

Populations differed from each other also with respect to mean behaviours and SMR. SG fish were on average bolder (emerged faster from a refuge), more active (shorter time spent being inactive and a non-significant trend was also observed for longer distance travelled), and had higher mass-specific SMRs than fish from the FG population (Table 1 and Table S3 in SI). However, SG fish were found more often within the refuge area than FG fish. Mean differences in behaviour were maintained between populations whether or not we accounted for the effect of different body sizes (Fig. S5 in SI). Notably, SG fish (on average larger than FG ones) showed a higher SMR than FG ones only after the hidden contribution of variation in body size was accounted for, that is, when comparisons referred to a standard body size (Fig. S5 in SI).

Within populations, hiding time, distance moved, mass-specific SMR, and standard size were repeatable over ontogeny (Table 2). On the contrary, emergence latency and freezing time were not repeatable (data not shown) and, thus, were excluded from subsequent analyses. Moreover, distance moved decreased with age in FG fish, but not in SG fish (Table 2). FG males were smaller and showed higher mass-specific SMRs than their female counterpart, while no differences in standard size and mass-specific SMR were present between sexes in the SG population (Table 2). Nevertheless, males and females did not differ in their average behaviours in both populations (Table 2).

**Table 2:** Parameter estimates (±SE) of fixed and random effects derived from univariate models fitted to partition variation in hiding time (i.e., boldness), distance moved (i.e., activity), standard size, and mass-specific SMR with respect to SG and FG fish.

Fixed effects	SG				FG			
	Hiding time	Distance moved	Size	SMR	Hiding time	Distance moved	Size	SMR
	β (SE)	β (SE)	β (SE)	β (SE)	β (SE)	β (SE)	β (SE)	β (SE)
Intercept	0.314 (0.262)	**0.903 (0.241)**	0.186 (0.115)	−0.049 (0.149)	**−0.825 (0.226)**	0.181 (0.223)	**−0.108** **(0.030)**	−0.018 (0.149)
Age	0.168 (0.158)	0.093 (0.136)	**1.389 (0.058)**	**−1.536 (0.077)**	0.113 (0.134)	**−0.481 (0.131)**	**1.820 (0.046)**	**−1.475** **(0.088)**
Sex	−0.235 (0.228)	−0.081 (0.260)	−0.263 (0.234)	0.237 (0.190)	0.139 (0.206)	0.351 (0.207)	**−0.440 (0.060)**	**0.438** **(0.142)**
Trial	−0.077 (0.158)	**−0.613 (0.136)**	—	—	**0.452 (0.134)**	−0.138 (0.131)	—	—
**Random effects**	**σ² (SE)**	**σ² (SE)**	**σ² (SE)**	**σ² (SE)**	**σ² (SE)**	**σ² (SE)**	**σ² (SE)**	**σ² (SE)**
Individual	0.196 (0.106)	0.361 (0.134)	0.382 (0.107)	0.222 (0.071)	0.226 (0.096)	0.240 (0.097)	0.013 (0.008)	0.114 (0.045)
Residual	0.775 (0.115)	0.575 (0.085)	0.106 (0.016)	0.185 (0.027)	0.721 (0.094)	0.691 (0.090)	0.085 (0.011)	0.300 (0.040)
Repeatability	**0.202 (0.097)**	**0.386 (0.100)**	**0.783** **(0.055)**	**0.546 (0.090)**	**0.238 (0.086)**	**0.258 (0.087)**	**0.137** **(0.080)**	**0.275** **(0.089)**

Because of the nature of the variable, a low hiding time corresponds to a high boldness score. Random effects are expressed as the proportion of total phenotypic variation not attributable to fixed effects. Values printed in bold represent significant effects based either on Wald F tests (for fixed effects) or LRTs (for random effects).

**Figure 2:**
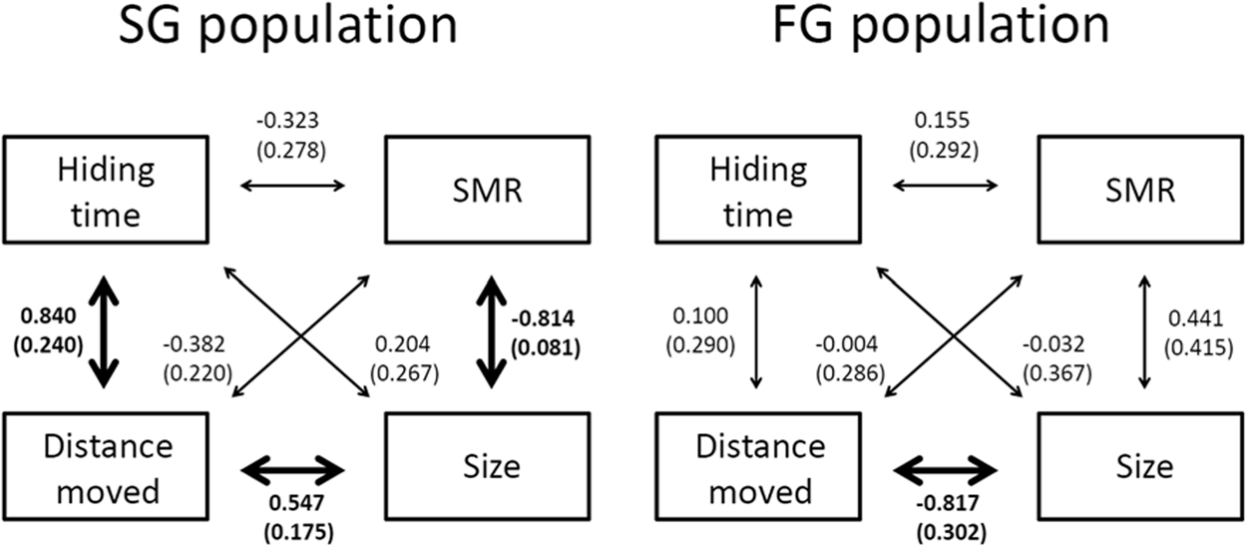
Estimated among-individual correlations (with SE) between phenotypic traits. Phenotypic traits represented here were repeatable over the ontogeny for both SG and FG fish, that is, hiding time (i.e., boldness), distance moved (i.e., activity), mass-specific SMR, and standard size. Because of the nature of the variable, a low hiding time corresponds to a high boldness score. Correlations printed in bold are significant (*P* < 0.05) based on LRTs derived from the multivariate model as detailed in the main text.

Within the SG population, we found evidence for among-individual correlations between hiding time, distance moved, mass-specific SMR, and standard size as predicted by POLS (Fig. 2). Specifically, larger SG fish were more active and had lower mass-specific SMRs compared to their smaller siblings, while hiding time and distance moved were positively correlated (Fig. 2; Table S4 in SI). In contrast, we did not find statistically significant correlations among POLS traits across individuals from the FG population, except for the tendency of larger fish to travel shorter distances than their smaller siblings (Fig. 2; Table S4 in SI).

Overall, pairwise covariances between traits differed significantly between SG and FG fish (*P* < 0.001; χ^2^ = 38.891, df = 6), confirming that the two populations differed from each other in POLS. Moreover, comparisons of full and constrained models revealed that covariances were overall significant in the SG (*P* < 0.001; χ^6^ = 40.25, df = 6) but not in the FG (*P* = 0.25; χ^6^ = 7.83, df = 6) population, confirming that POLS was present only in SG fish.

Notably, most within-individual (i.e., residual) correlations were not significant in either populations, except for the expected negative correlation between mass-specific SMR and fish size (i.e., the larger the individual is, the lower is its mass-specific SMR; Table S4 in SI).

## Discussion

We tested predictions from the pace-of-life syndrome (POLS) hypothesis on offspring from two distinct fish populations whose native environments differed in abiotic and biotic factors. We found that populations differed on average in their life history, thus confirming variable investments in survival and reproduction between populations. Yet, contrary to expectations, fish from the slow-growing population (SG) were on average bolder (lower emergence latency), more active (shorter time spent being inactive and a non-significant trend was also observed for longer distance travelled), and had higher mass-specific metabolic costs for their body maintenance than fish from the fast-growing population (FG). Within populations, most life-history, behavioural, and physiological traits were repeatable over ontogeny. However, among-individual correlations between these traits were found mostly in SG, but less in FG fish. As a result, POLS was overall present in SG but not FG fish, with correlation matrices also differing between populations, which confirmed divergence in the occurrence of POLS.

Laboratory-reared offspring obtained from two geographically isolated populations differed on average in their life-history strategy despite identical housing conditions, indicating that differences were not generated by plastic responses to the environment over ontogeny. Nevertheless, populations reported here represent only two independent data points and their native environments differed in a variety of biotic and abiotic factors. Therefore, we cannot separate the effect of a specific selection force (e.g., size-related mortality^54^ or temperature fluctuations^55^) from the contribution of other factors (e.g., genetic drift^56^) on different life histories observed here. Whether those differences in life histories were genetically inherited or generated via parental effects is beyond the interest of this study.

A hallmark in POLS hypothesis is that life history trade-offs (survival *vs* reproduction) explain why consistent among-individual variation in behaviour and physiology is maintained within animal populations (“state-dependent personality”)^1,3,57^. The independent evolution of correlated traits should then be constrained, whereby pleiotropic effects of genes and common physiological pathways^19^ or correlational selection^58^ constrain changes in one trait (e.g., boldness) because of simultaneous selection on a correlated trait (e.g., growth rate^4^). Recent studies, however, have found mixed support for predictions from POLS (see reviews by^12–14,59^). Our findings contribute to the current debate^26^ by suggesting that the evolution of POLS within populations may depend on specific ecological conditions. We observed, indeed, that POLS differed substantially between our two fish populations inhabiting very diverse ecological contexts, whereby POLS was overall present in the SG but not in the FG population. In particular, those SG individuals with a larger body size at a given age exhibited higher activity levels^4^ (longer distance travelled) and higher absolute SMRs^17,60^ (i.e., lower mass-specific SMRs) compared to their smaller siblings, in agreement with general predictions from^1^. SG fish that travelled longer distances were also observed more often within the refuge area, suggesting that a behavioural syndrome was also present^19^. Nevertheless, trait correlations were absent among FG individuals (except one out of six trait combinations). In this direction, modelling approaches have recently stressed the importance of variation in environmental conditions as drivers for variation in POLS within species^28^. Here, our highly-standardized setup allowed to control for environmental effects that can typically mask correlations among POLS traits through individual plasticity (see^26^ and references therein). Thus, patterns of among-individual covariance observed here were generated by either genetic correlations and/or irreversible plasticity^26^. Whether variation in POLS traits observed here under standardized conditions reflect the full variation expressed by fish in the wild is unknown (see^61^ and references therein). Nevertheless, growing evidence suggest that among-individual variation in POLS traits is maintained consistently across laboratory and wild settings when individuals are measured repeatedly in both contexts^62,63^. The mismatch between environmental conditions experienced by fish in the lab and in their natural habitat (e.g., temperature regimes) should have therefore not altered the expression of POLS in the short-term, supporting the idea that variation in POLS observed here between populations was not caused by reversible plastic responses. It is worth noting that we had sufficient statistical power to detect repeatable among-individual differences in POLS traits over ontogeny in both populations of mosquitofish. Therefore, the lack of correlation between traits in FG fish cannot be attributed to low statistical power, as also observed in^20^ with a comparable sample size.

Ecological conditions might not only shape LHTs, and behavioural and physiological traits respond accordingly, but correlations within the entire suite of traits might be adaptively modulated by the environment^27,29,30,57^. Modelling approaches indicate variation in ecological conditions such as mortality risk to play a critical role in favouring or breaking apart combinations between POLS traits^28^. In agreement with predictions, empirical evidence confirmed that patterns of behavioural correlations varied among fish populations^20,21^, including mosquitofish^64^, adapted to divergent predator regimes. Different correlations among POLS traits observed here between SG and FG fish could then be explained as the result of variation in mortality risks in their native environment. FG fish have suffered from a strong selection primarily against large body sizes, whereas the survival for smaller FG fish in the wild did not depend on other components of an individual’s phenotype such as boldness or energy metabolism. The lack of correlational selection^58^ between those traits might have then relaxed constrains and favoured their independent evolution in FG fish. Opposite correlations between activity (distance moved) and size-at-age between SG and FG fish might also suggest that differing trait combinations can be adaptive under variable contexts. POLS predicts higher activity rates in larger fish, but lower distance moved observed in larger FG fish might have adaptively contrasted their high vulnerability to harvesting^65^, which is also confirmed by a large reduction in their distance moved over the lifetime. A second explanation for the lack of POLS in FG fish is that behavioural traits not measured in this study (e.g., aggressiveness) act as mediators of life history trade-offs for the FG population^1,3,26^. A short life expectancy in FG fish might have favoured aggressiveness over boldness in mediating access to resources in the absence of predators, other than humans. However, neither aggressiveness nor other risk-taking behaviours mediated life history trade-offs in Mediterranean field crickets^66^ (*Gryllus bimaculatus*, De Geer 1773), suggesting that the association between life-history and behavioural traits was likewise weak in crickets, in contrast to classic predictions by POLS. Lastly, it is also possible that diverse correlation patterns in POLS traits observed here arose in response to different climatic conditions experienced by fish in their native environments (see^67^). In their review, Hille and collaborators^12^ have indeed provided convincing evidence that relationships between life history, behaviour, and physiology may vary among animals across climatic gradients. Similarly, recent model results have suggested temperature variation to be the driver of correlations among POLS traits in animal populations^68^. Nevertheless, our limited knowledge on ecological conditions within native environments of the two populations measured here and the lack of replication does not allow us in favouring one explanation over others.

When comparing the two populations, we observed that slower life histories (SG) were on average coupled with bolder behaviours (faster emergence from a refuge), higher activity rates (lower freezing and a non-significant trend was also observed for longer distance travelled), and higher metabolic costs compared to faster life histories (FG). This result also contrasts with the concept of fast and slow POLS, whereby fast life histories are predicted to match risky behaviours and higher metabolic rates^8^. Energetic trade-offs^28^ might instead force FG individuals with higher growth rates to decrease energetically costly behaviours and metabolic demands in contrast to SG ones^69^. In support of this interpretation, we observed that diverse swimming rates observed between populations in the open field matched diverse “routine” swimming rates observed in their housing conditions (Fig. S6 in SI), thus expanding mean behavioural differences observed here beyond the open field test.

Since the POLS hypothesis has been formulated^1^, most empirical studies have concentrated on confirming its existence across the animal kingdom. Empirical support remains nevertheless ambiguous^13,14^ and POLS is currently subject to hot debate^26^. Our results suggest that ecological conditions might affect the evolution of POLS in different populations of the same species, as already unambiguously verified for the evolution of life-history^70^ and behavioural strategies^21^. Building on this evidence, we suggest that future studies with replicated populations would allow the role of different selection pressures in shaping the correlation structure of POLS traits to be disentangled. This would enable predictions put forth by POLS to provide specific predictions concerning the strength and sign of trait correlations across ecological gradients, and ultimately explain the maintenance of their among-individual variation.

## Electronic supplementary material

Supplementary Information
