# Supplementary material for: Ecological conditions drive pace-of-life syndromes by shaping relationships between life history, physiology and behaviour in two populations of Eastern mosquitofish
Source: Sci Rep. 2018 Oct 2;8:14673. doi: 10.1038/s41598-018-33047-0 (PMC6168454; doi:10.1038/s41598-018-33047-0)
Supplement: Supplementary file 1 — Supplementary Information [file 41598_2018_33047_MOESM1_ESM.pdf]

Ecological conditions drive pace-of-life syndromes by shaping relationships between life history, physiology and behaviour in two populations of Eastern mosquitofish

Giovanni Polverino, Francesca Santostefano, Carlos Díaz-Gil, and Thomas Mehner

Supplementary Information (SI)

Methods

1. *Behavioural assays*

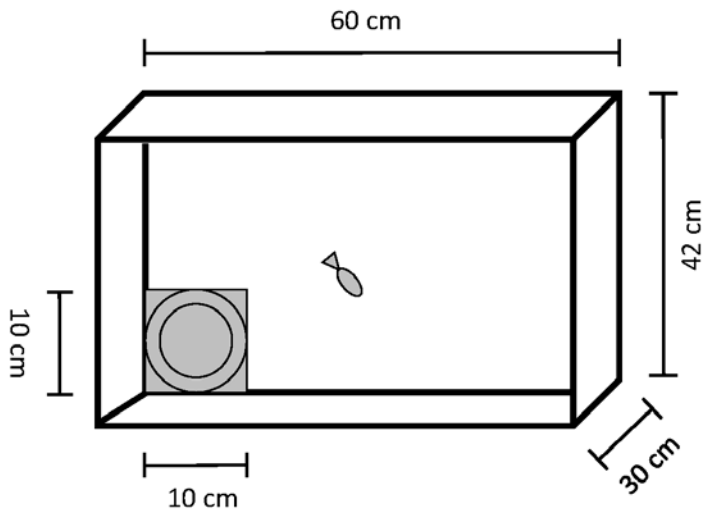

Figure S1. Top-view schematic of the open field. The two concentric circles represent the refuge with its sliding lid (used for acclimating the fish before the test), while the grey-squared area around the closed refuge represents the only shelter available to the fish during the test. Both hiding area and refuge are here illustrated as grey to facilitate their identification but they were both coloured in white, consistently with the background of the open field.

2. *Standard metabolic rate*

A computerized intermittent flow respirometer (DAQ-PAC-WF8, Loligo Systems, Tjele, Denmark, www.loligosystems.com) was utilized to perform metabolic measurements. An array of eight cylindrical glass chambers (5 mL each) was utilized to test seven fish simultaneously, while a chamber was left unoccupied and used to measure bacterial respiration. Chambers were submerged in an aerated water bath maintained at a constant temperature of 23 ± 0.1°C, consistent with the water temperature that

experimental fish experienced in both housing tanks and open field. An ultraviolet sterilizer (EHEIM reeflex-UV-350, EHEIM GmbH, Germany, www.eheim.com) was connected to the water bath through a closed recirculation system to maintain low bacteria concentrations in the water during night. Water oxygen content within chambers was measured once every second using eight independent oximeter sensors (Firesting 8-Channel oxygen meters, Pyroscience, Aachen, Germany, www.pyro-science.com). The entire apparatus was located within a second temperature-controlled room, which remained closed and undisturbed during measurements.

The oxygen-saturated water from the water bath was periodically flushed into each chamber of the respirometer through an external pump that was set to turn on and off for alternating 10 min periods. After 1 min since the flow of oxygen-saturated water into the chambers was interrupted (i.e., waiting phase), the decrease in oxygen content in the closed chambers was measured once every second for a 9 min period (i.e., closed phase). Subsequently, each chamber was automatically flushed with aerated water for 10 min (i.e., flushing phase) before starting the next measurement. A second external pump sustained a slow recirculation during the experiment to guarantee uniform oxygen levels within each chamber of the respirometer.

3. Life-history assessment

Body shape of each individual was analysed using the geometric morphometry-landmarks method[1]. Eleven (males) and ten (females) different landmarks were selected similarly to previous studies on mosquitofish[2]. As described in detail by[2], fixed landmarks were chosen to characterize especially the region of the fish tail devoted to locomotor performances (i.e., swimming muscle, mm^2), according to the area marked

between landmarks 4-8 (Figure S2). Gonopodium length (mm) was also measured in male fish and it was represented by the segment defined by landmarks 9 and 10 (Figure S2). The outline of female individuals was instead characterized by 10 landmarks only (i.e., absence of landmark 9).

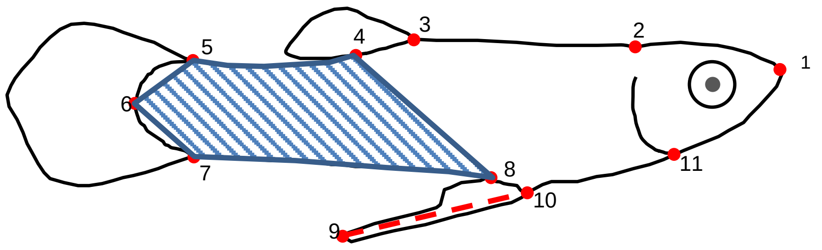

Figure S2. Outline of an adult male *Gambusia holbrooki* based on 11 fixed landmarks. Landmarks were defined as follows: 1, tip of the snout; 2, depression on the end of the head; 3 and 4, insertions of the dorsal fin; 5 and 7, insertions of the caudal fin; 6, end of the caudal muscle; 8 and 10, insertions of the anal fin; 9 (only in males), tip of the gonopodium; and 11, insertion of the operculum. Polygon in shade represents the "swimming muscle", following the description by[2]. The red dashed line represents the gonopodium length.

The xy coordinates of those landmarks were digitalized using tpsDig2 software[3] and superimposed using a Generalized Procrustes Analysis (GPA) as implemented in the function *procGPA* from the *shapes* library[4] of the R-3.1.1 software[5]. The superimposed landmarks were used as input for a MANOVA using redundancy analysis (RDA) to test for average differences in the total morphometry of adult individuals between the two populations. The test was performed on males and females separately since the number of landmarks varied between sexes. Furthermore, the individual size (i.e.,

standard length) was standardized to account for differences in size between individuals.

The total number of eggs and their dry weight (mg) were measured per each female separately with an electronic microscope (Modular stereo microscope MZ8, Leica Microsystems, Wetzlar, Germany, www.leica-microsystems.com) and a laboratory-weight scale (Secura 124-1CCH analytical balance, Sartorius AG, Goettingen, Germany, www.sartorius.com) respectively, while the mean dry weight per egg (mg) was estimated indirectly as the total number of eggs for a given female divided by their total dry weight (mg; Figure S3).

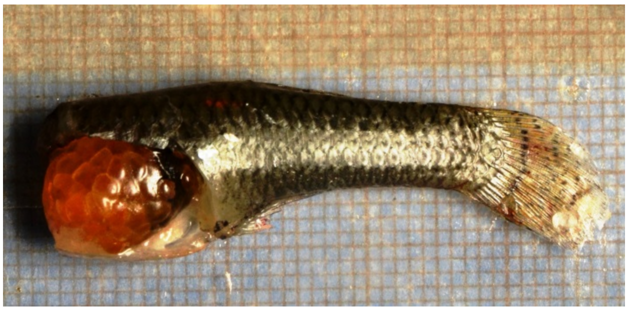

Figure S3. Snapshot of the abdominal cavity of an adult female *Gambusia holbrooki* examined for fecundity measurements (number of eggs, their dry weight, and mean dry weight per egg).

Results

1. Morphometric measurements

RDA results offered significant whole body shape differences between fish from the slow-growing (SG) and the fast-growing (FG) population in both males and females (F_1=3.2858, *P*= 0.033; and F_1=4.1952, *P*= 0.001, respectively; Figure S4).

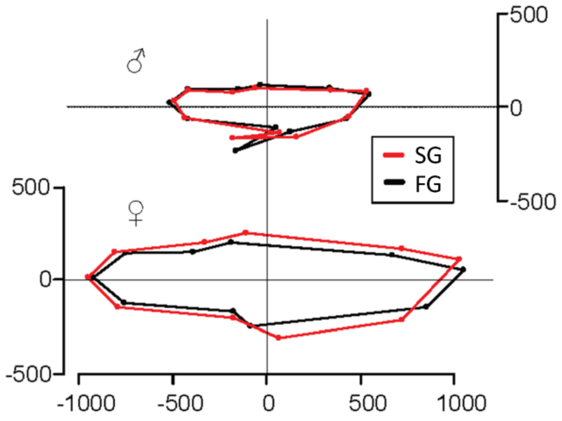

Figure S4. Schematic of the average body shape outlined for both males (above) and females (below) separately for each population (SG and FG). Differences are magnified (three times) to facilitate the graphical identification of the landmarks driving those differences. The units are based on the a-dimensional re-dimensioning carried out by the GPA analysis (the coordinate 0, 0 is the centroid centre). Notably, standard body size was standardized to favour a comprehensive interpretation of differences between populations and sexes.

2. *Mean differences in behaviour, mass-specific standard metabolic rate, and size-at-age between populations based on univariate models*

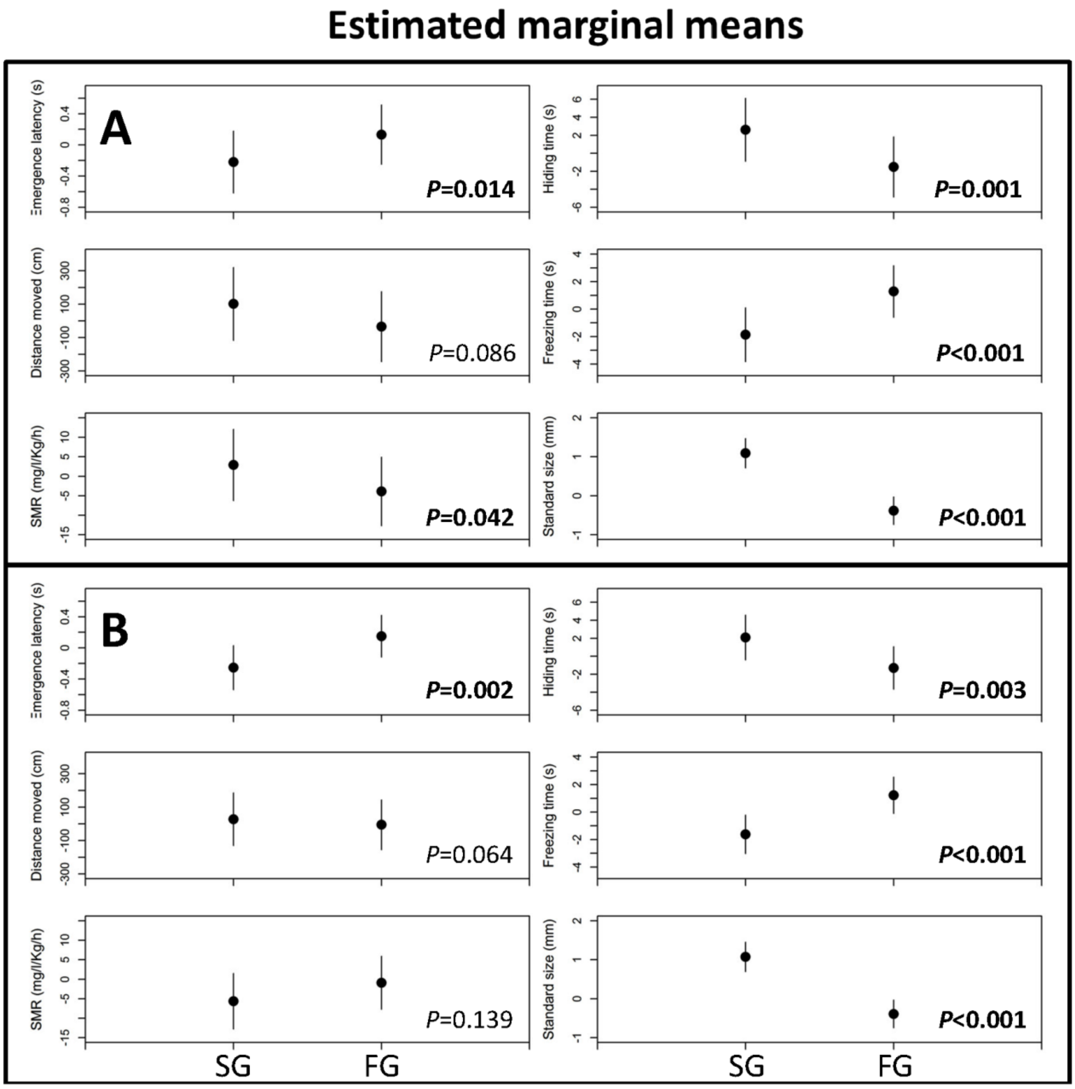

Figure S5. Estimated marginal means (± CI) with (A) and without (B) accounting for mean differences in body size between fish from the SG and FG population. Estimated marginal means represent adjusted means with respect to emergence latency and hiding time (boldness), distance moved and freezing time (activity), mass-specific standard metabolic rate (SMR), and standard size for each fish population, once the contribution from fixed effects (i.e., age, sex, and trial) is accounted for. Because of the nature of the variables, both short emergence latency and low hiding time correspond to a high boldness score.

3. Correlation between swimming rates (i.e., activity) in the open field and in the housing Plexiglas cylinder

Two weeks after the conclusion of the main experiment, we have selected three groups of six fish each (i.e., 18 fish total) that respectively spent the lowest, intermediate, and highest time swimming (i.e., swimming time, s) within the open field. Then, we have tested them again for their swimming time within their housing Plexiglas cylinders. As for the tests in the open field, the behaviour of each fish within its housing cylinders was measured across two 10-min trials, two days apart from each other. We ran a Pearson product-moment correlation to test whether swimming time in the open field and in the housing cylinders were correlated with each other, that is, whether among-individual differences in activity rates measured in the open field reflected different activity rates among fish within their housings. We observed that swimming time measured in the open field was a valid proxy for the routine swimming time observed in fish within their housing cylinders (Figure S6), thus expanding behavioural outcomes measured in this study outside of the open field test.

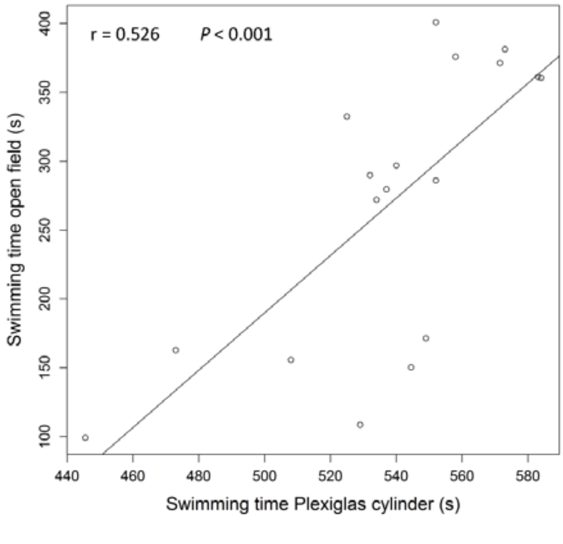

Figure S6. Correlation between swimming time in the open field and swimming time in the housing cylinder.

4. Univariate LMMs: correlations between pairs of fixed effects

We estimated the phenotypic correlation[6] (i.e., the overall correlation jointly contributed by among- and within-individual correlations) between each pair of LHTs measured. To do this, we used bivariate linear mixed-effects models[6] (LMMs) using Markov Chain Monte Carlo techniques, while including individuals as a random effect (i.e., random intercepts) to account for repeated measures of the same individuals over time. The bivariate LMMs were performed using MCMC sampling methods under a Bayesian framework (R package 'MCMCglmm'[7]). The parameters were estimated using a non-informative prior, with 1 500 000 resamplings, 500 000 burn-ins, and 100 thinnings.

Table S1. Phenotypic-correlation estimates between pairs of LHTs. The best estimate of correlation coefficients (i.e. values above the diagonal) and their 95% credible intervals (i.e. values below the diagonal) are represented for each pair of LHTs. Significant results are represented in bold and correspond to correlation coefficients whose confidence intervals do not overlap with zero.

	Standard size	Fulton's K	Body weight
Standard size	-	-0.065	**0.597**
Fulton's K	-0.155<br>0.029	-	0.058
Body weight	**0.521**<br>**0.669**	-0.099<br>0.219	-

5. Univariate LMMs that include mother ID (i.e., family) and tank as fixed effects

Table S2. Parameter estimates (± SE) of fixed and random effects derived from univariate models fitted to partition variation in hiding time (i.e., boldness), distance moved (i.e., activity), standard size (size), and mass-specific SMR with respect to SG and FG fish. Because of the nature of the variable, a low hiding time corresponds to a high boldness score. Random effects are expressed as the proportion of total phenotypic variation not attributable to fixed effects. Values printed in bold represent significant effects based either on Wald F tests (for fixed effects) or LRTs (for random effects). [a]Reference is mother 1.

	SG				FG			
	Hiding time	Distance moved	Size	SMR	Hiding time	Distance moved	Size	SMR
Fixed effects	β (SE)	β (SE)	β (SE)	β (SE)	β (SE)	β (SE)	β (SE)	β (SE)
Intercept	0.264 (0.341)	**0.794 (0.336)**	0.338 (0.252)	-0.152 (0.232)	**-0.950 (0.244)**	-0.073 (0.238)	**-0.072 (0.041)**	0.160 (0.159)
Age	0.168 (0.158)	0.093 (0.136)	**1.389 (0.058)**	**-1.536 (0.077)**	0.113 (0.134)	**-0.481 (0.131)**	**1.820 (0.046)**	**-1.486 (0.089)**
Sex	-0.297 (0.245)	-0.202 (0.267)	-0.245 (0.252)	0.161 (0.201)	0.156 (0.206)	0.229 (0.201)	**-0.406 (0.061)**	**0.538 (0.135)**
Mother 2[a]	0.343 (0.392)	0.671 (0.427)	-0.032 (0.403)	0.407 (0.321)	0.000 (0.000)	0.000 (0.000)	0.000 (0.000)	0.000 (0.000)
Mother 3[a]	0.000 (0.000)	0.000 (0.000)	0.000 (0.000)	0.000 (0.000)	-0.546 (0.305)	0.095 0.298	-0.137 0.090	-0.046 0.200
Tank	-0.005 (0.287)	0.008 (0.314)	-0.240 (0.296)	0.064 (0.235)	0.629 (0.286)	**0.466 0.279**	0.017 0.085	**-0.354 0.187**
Trial	-0.077 (0.158)	**-0.613 (0.136)**	-	-	**0.452 0.134**	-0.138 (0.131)	-	-
Random effects	σ^2 (SE)	σ^2 (SE)	σ^2 (SE)	σ^2 (SE)	σ^2 (SE)	σ^2 (SE)	σ^2 (SE)	σ^2 (SE)
Individual	0.207 (0.112)	0.333 (0.132)	0.399 (0.116)	0.223 (0.073)	0.197 (0.092)	0.187 0.088	0.012 (0.008)	0.079 (0.039)
Residual	0.775 (0.115)	0.575 (0.085)	0.106 (0.016)	0.185 (0.027)	0.721 0.094	0.691 (0.090)	0.085 (0.011)	0.304 (0.040)
Repeatability	**0.211 (0.099)**	**0.367 (0.103)**	**0.790 (0.055)**	**0.547 (0.093)**	**0.214 0.087**	**0.213 (0.087)**	**0.122 (0.081)**	**0.206 (0.090)**

6. Univariate LMMs with population, age, sex, standard size, Fulton's K, and trial included as fixed factors and the individual included as a random effect

Table S3. Parameter estimates (± SE) of fixed effects derived from univariate models fitted to partition variation in emergence latency and hiding time (i.e., boldness), distance moved and freezing time (i.e., activity), and mass-specific standard metabolic rate (i.e., SMR). Values printed in bold face represent significant effects based on Wald F tests.

	Emergence latency	Hiding time	Distance moved	Freezing time	SMR
Fixed effects	β (SE)	β (SE)	β (SE)	β (SE)	
Intercept	-0.109 (0.237)	-2.972 (2.068)	200.76 (127.94)	-0.927 (1.166)	**16.46 (5.20)**
Population	**0.358 (0.148)**	**-4.256 1.343**	-23.74 (86.23)	**3.037 (0.728)**	**-7.089 (3.608)**
Age	0.028 (0.297)	4.770 (2.596)	162.66 (158.00)	-2.637 (1.463)	**-25.37 (6.224)**
Sex	-0.024 (0.156)	-0.454 (1.438)	45.75 (94.21)	0.134 (0.768)	1.400 (3.969)
Standard size	-0.029 (0.046)	-0.580 (0.409)	**-55.36 (24.99)**	0.141 (0.229)	**-7.277 (0.993)**
Fulton's *K*	0.710 (0.454)	4.169 (4.015)	**557.08 (247.93)**	3.853 (2.235)	**-54.78 (9.853)**
Trial	-0.080 (0.121)	**2.341 (1.029)**	**-191.55 (60.61)**	0.251 (0.600)	-

7. *Covariances and correlations between repeatable traits for each population*

Table S4. Estimated among- (A) and within- (B) individual covariances and correlations (± SE) between hiding time (i.e., boldness), distance moved (i.e., activity), standard size, and standard metabolic rate (SMR) in SG and FG fish. We present covariances (lower-off diagonals) and correlations (upper-off diagonals) for each set of traits. Because of the nature of the variable, a low hiding time corresponds to a high boldness score. Correlations printed in bold-face are significant (*P*<0.05) based on likelihood ratio tests derived from the multivariate model detailed in the main text.

		SG				FG			
		Hiding time	Distance moved	Size	SMR	Hiding time	Distance moved	Size	SMR
A	Hiding time	-	**0.840 (0.240)**	0.204 (0.267)	-0.323 (0.278)	-	0.100 (0.290)	-0.032 (0.367)	0.155 (0.292)
	Distance moved	**0.223 (0.093)**	-	**0.547 (0.175)**	-0.382 (0.220)	0.023 (0.069)	-	**-0.817 (0.302)**	-0.004 (0.286)
	Size	0.056 (0.075)	**0.203 (0.093)**	-	**-0.814 (0.081)**	-0.002 (0.020)	**-0.046 (0.022)**	-	0.441 (0.415)
	SMR	-0.067 (0.062)	-0.108 (0.071)	**-0.237 (0.078)**	-	0.024 (0.046)	-0.001 (0.046)	0.017 (0.014)	-
B	Hiding time	-	-0.058 (0.104)	-0.042 (0.105)	0.040 (0.105)	-	0.149 (0.090)	0.046 (0.092)	-0.073 (0.092)
	Distance moved	-0.039 (0.070)	-	-0.003 (0.105)	0.077 (0.104)	0.105 (0.066)	-	-0.053 (0.092)	0.028 (0.093)
	Size	-0.012 (0.030)	-0.001 (0.026)	-	**-0.461 (0.083)**	0.011 (0.023)	-0.013 (0.022)	-	**-0.483 (0.071)**
	SMR	0.015 (0.040)	0.025 (0.034)	**-0.065 (0.016)**	-	-0.034 (0.043)	0.013 (0.043)	**-0.078 (0.017)**	-

References

1 Rohlf, F. J. & Marcus, L. F. A revolution morphometrics. *Trends in Ecology & Evolution* **8**, 129-132 (1993).

2 Langerhans, R. B., Layman, C. A., Shokrollahi, A. & DeWitt, T. J. Predator-driven phenotypic diversification in *Gambusia affinis*. *Evolution* **58**, 2305-2318 (2004).

3 Rohlf, F. J. tpsdig2 Stony Brook. *NY: Department of Ecology and Evolution, State* (2004).

4 Dryden, I. & Dryden, M. I. Shapes package. *Vienna, Austria: R Foundation for Statistical Computing* (2012).

5 R Core Team. R: A language and environment for statistical computing. *R Foundation for Statistical Computing* (2013).

6 Dingemanse, N. J. & Dochtermann, N. A. Quantifying individual variation in behaviour: mixed-effect modelling approaches. *Journal of Animal Ecology* **82**, 39-54 (2013).

7 Hadfield, J. D. MCMC methods for multi-response generalized linear mixed models: the MCMCglmm R package. *Journal of Statistical Software* **33**, 1-22 (2010).
